# NGS-Based Diagnosis of Treatable Neurogenetic Disorders in Adults: Opportunities and Challenges

**DOI:** 10.3390/genes12050695

**Published:** 2021-05-06

**Authors:** Jean-Marc Good, Isis Atallah, Mayte Castro Jimenez, David Benninger, Thierry Kuntzer, Andrea Superti-Furga, Christel Tran

**Affiliations:** 1 Division of Genetic Medicine, Lausanne University Hospital (CHUV), 1011 Lausanne, Switzerland; jean-marc.good@chuv.ch (J.-M.G.); Maria-Isis.Atallah-Gonzalez@chuv.ch (I.A.); andrea.superti-furga@chuv.ch (A.S.-F.); 2 Division of Neurology, Lausanne University Hospital (CHUV), 1011 Lausanne, Switzerland; Mayte.Castro-Jimenez@chuv.ch (M.C.J.); david.benninger@chuv.ch (D.B.); thierry.kuntzer@chuv.ch (T.K.)

**Keywords:** neurogenetic disorders, cerebrotendinous xanthomatosis, X-linked adrenoleukodystrophy, glucose transporter type 1 deficiency syndrome, treatable diseases, next-generation sequencing (NGS)

## Abstract

The identification of neurological disorders by next-generation sequencing (NGS)-based gene panels has helped clinicians understand the underlying physiopathology, resulting in personalized treatment for some rare diseases. While the phenotype of distinct neurogenetic disorders is generally well-known in childhood, in adulthood, the phenotype can be unspecific and make the standard diagnostic approach more complex. Here we present three unrelated adults with various neurological manifestations who were successfully diagnosed using NGS, allowing for the initiation of potentially life-changing treatments. A 63-year-old woman with progressive cognitive decline, pyramidal signs, and bilateral cataract was treated by chenodeoxycholic acid following the diagnosis of cerebrotendinous xanthomatosis due to a homozygous variant in *CYP27A1*. A 32-year-old man with adult-onset spastic paraplegia, in whom a variant in *ABCD1* confirmed an X-linked adrenoleukodystrophy, was treated with corticoids for adrenal insufficiency. The third patient, a 28-year-old woman with early-onset developmental delay, epilepsy, and movement disorders was treated with a ketogenic diet following the identification of a variant in *SLC2A1*, confirming a glucose transporter type 1 deficiency syndrome. This case study illustrates the challenges in the timely diagnosis of medically actionable neurogenetic conditions, but also the considerable potential for improving patient health through modern sequencing technologies.

## 1. Introduction

Neurogenetic diseases represent a complex group of Mendelian disorders affecting the central or peripheral nervous system with varied clinical presentations. For the majority of them, therapeutic options are limited and mainly supportive [1]. However, progress in the understanding of the underlying physiopathology has led to the development of treatments, including dietary interventions, enzyme replacement therapy, specific medications, and others, which may remarkably improve the clinical course [2,3]. Currently, a large proportion of these treatable conditions consist of inborn errors of metabolism (IEM). A well-known example is phenylalanine hydroxylase deficiency, namely phenylketonuria (PKU, OMIM 261600), in which a low phenylalanine diet implemented after birth prevents the development of irremediable intellectual disability [4]. As in PKU, the benefit of therapeutic interventions in other treatable neurogenetic disorders is often larger when provided early in the clinical course, averting irreversible damages to the nervous system [2]. Although most of the IEM present soon after birth or during childhood, an increased number of late-onset forms presenting in adulthood are now known [5].

A prompt and accurate diagnosis is essential to providing the patient with potentially life-changing treatment, as well as genetic counseling. In this regard, next-generation sequencing (NGS) has provided new possibilities for the investigation of neurological patients in combination with clinical data, neuroimaging, and laboratory findings. Nevertheless, the diagnostic process remains challenging, notably due to the complexity of the field, with a large amount of extremely rare and recently discovered conditions [6].

Here, we document three unrelated adult patients in whom specific treatments were provided after being diagnosed with cerebrotendinous xanthomatosis (CTX) (OMIM 213700), X-linked adrenoleukodystrophy (X-ALD) (OMIM 300100), and glucose transporter type 1 deficiency syndrome (Glut1 DS) (OMIM 606777).

## 2. Materials and Methods

### Molecular Genetic Testing

Genomic DNA was isolated from leukocytes and whole-exome sequencing (WES) was carried out on an Illumina HiSeq 2500 sequencer (Illumina, Inc., San Diego, CA, USA) with an Agilent SureSelectXT Human All Exon V7 capture kit (Agilent Technologies, Santa Clara, CA, USA). Data processing, variants annotation, and filtering were performed as previously described [7]. We analyzed a virtual panel (hereditary spastic paraplegia, 147 genes) in patient 2, and the *CYP27A1* and *SLC2A1* genes in patients 1 and 3, respectively. Identified variants were interpreted according to the American College of Medical Genetics and Genomics (ACMG) variant classification guidelines [8]. They were subsequently confirmed by Sanger sequencing. Segregation analysis by Sanger sequencing was performed in the parents of proband 3.

## 3. Results

### 3.1. Proband 1

**Figure 1:**
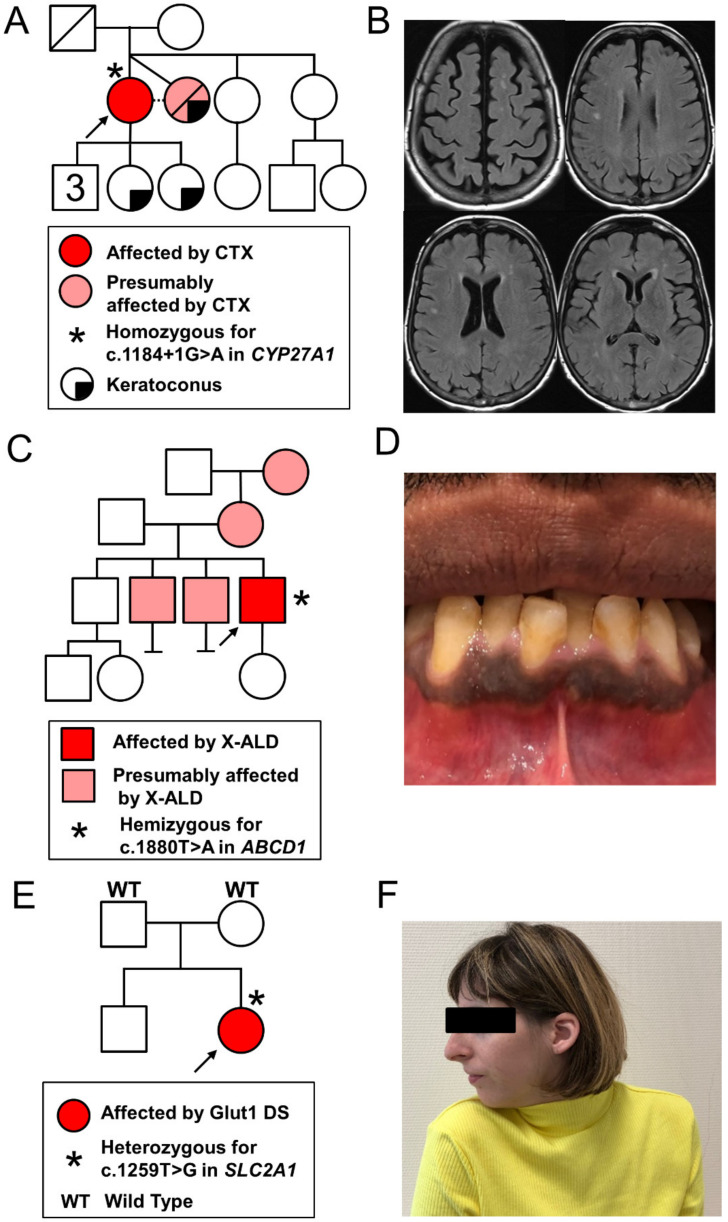
Family trees and phenotypic data of the three patients: (**A**,**B**) proband 1: family pedigree (left panel) and axial views of brain MRI, FLAIR images showing non-confluent bilateral periventricular and semi-oval center white matter hyperintensities (right panel); (**C**,**D**) proband 2: family pedigree (left panel) and a picture of the mouth showing gingival hyperpigmentation (right panel); (**E**,**F**) proband 3: family pedigree (left panel) and a photograph showing cervical dystonia with right torticollis, as well as microcephaly. CTX, cerebrotendinous xanthomatosis; Glut1 DS, glucose transporter type 1 deficiency syndrome; X-ALD, X-linked adrenoleukodystrophy.

A 61-year-old woman presented with an 8-year history of progressive spastic tetraparesis, predominantly affecting lower extremities, and cerebellar ataxia, causing walking difficulties. Subsequently, cognitive decline, including severe attention deficit, dysexecutive syndrome, and memory loss, was documented. She also experienced chronic diarrhea, which caused a 10 kg weight loss over 10 years. The review of her medical history revealed that she underwent bilateral cataract surgery at age 44 and that she developed a bilateral sensorineural hearing loss. She was born to healthy parents not known to be consanguineous. She had a twin sister (unknown zygosity) who developed a similar progressive phenotype, including cognitive dysfunction, cerebellar ataxia, spastic tetraparesis, and cataract from age 45, before her death from a cardiac disease at age 57 (Figure 1A). The diagnosis of multiple sclerosis was suspected.

Brain MRI showed bilateral hyperintensities of the periventricular and semi-oval centers white matter (Figure 1B). Furthermore, a nerve conduction study revealed a length-dependent sensorimotor axonal polyneuropathy of lower limbs. Genetic testing excluded trinucleotide repeat disorders including spinocerebellar ataxias (SCA) [1,2,3,6,7,9], Friedreich ataxia, dentatorubral-pallidoluysian atrophy, and fragile X-associated tremor/ataxia syndrome. Finally, the metabolic workup revealed increased levels of blood bile acids (25 μmol/L; normal value (n.v.) 0–10) and of cholestanol (59.99 μmol/L; n.v. 0–15.45) compatible with the diagnosis of cerebrotendinous xanthomatosis (CTX). CTX was confirmed by the identification of the pathogenic splice-site variant c.1184 + 1G>A in homozygosity in intron 6 of the *CYP27A1* gene.

Following the diagnosis, a focused clinical examination confirmed the absence of tendinous xanthomas. Treatment with chenodeoxycholic acid was introduced, and at the one-year follow-up, her blood cholestanol levels had normalized (13.3 μmol/L; n.v. 0–15.45). The diarrhea had disappeared, she had gained 4 kg, and a mild improvement in her cognitive function was found.

### 3.2. Proband 2

A 32-year-old man presented with a 5-year history of stiffness, spasms, and paresthesia of lower limbs, with progressive gait difficulties and occasional falls. He also experienced urinary urgencies and incontinence, as well as sexual dysfunction. Neurological examination revealed a tetrapyramidal syndrome predominantly affecting his lower extremities. The proband had three older brothers, two of which experienced similar but even more severe neurological symptoms (Figure 1C). His mother may have had (phasic) cervical dystonia and the maternal grandmother was reported to have had late onset stiffness and gait difficulties.

A nerve conduction study and brain and spine MRI were normal. The analysis of the WES data focused on a hereditary spastic paraplegia gene panel (147 genes) allowed the identification of the pathogenic variant c.1880T>A (p.Leu627His) in hemizygosity in the *ABCD1* gene, confirming the diagnosis of X-ALD. Elevated plasmatic very-long-chain fatty acids (VLCFA) corroborated this diagnosis (C24/22: 1.68; n.v. 0.3–1.1, C26/22: 0.075; n.v. 0.002–0.025 and C26: 2.8 μmol/L; n.v. 0.3–1.9). The clinical phenotype was suggestive of an adrenomyeloneuropathy (AMN), a typical adult-onset presentation of X-ALD.

Addison’s disease, being typically associated with AMN, informed a subsequent clinical evaluation of the patient, looking more specifically for symptoms and signs of adrenocortical insufficiency, which indeed revealed chronic fatigue, difficulty with weight gain, cravings for salty food, and orthostatic hypotension. Gingival hyperpigmentation was observed (Figure 1D). Addison’s disease was confirmed by documenting an elevated plasmatic adrenocorticotropic hormone (ACTH) (167 ng/L, n.v. 9–50) and an abnormal response to ACTH stimulation (cortisol level 1 h post i.v. injection of synacthen (0.25 mg): 353 nmol/L, n.v. > 500). Hormone replacement therapy with glucocorticoids (hydrocortisone) and mineralocorticoids (fludrocortisone) was initiated. An emergency card for Addison’s disease was given to the patient. Along with a genetic counselling, a family information letter was provided to the patient to give his siblings the opportunity to be evaluated at our clinic given their symptoms suggestive of AMN.

### 3.3. Proband 3

The proband was a 28-year-old woman born to healthy, unrelated parents (Figure 1E). Hypotonia and progressive microcephaly were observed during the first months of life. Her development was delayed (independent walking at age 2.5 years and language acquisition at 4 years) and she received special education. From age 2, various movement disorders including dystonic postures of fingers, dyskinesia, myoclonus, ataxia, and spasticity of lower limbs were reported. During this period, she also developed epilepsy with absence seizures and was treated with valproate. In addition, the patient presented with paroxysmal episodes of variable semiology including stiffness of the right hemibody, or hypotonia, causing difficulty walking, apathy, headache, nausea and vomiting, followed by somnolence.

Extensive investigations performed in the first years of life did not produce a diagnosis. In particular, a cerebral MRI at age 1 year revealed non-specific cortical white matter anomalies. Her karyotype and the methylation of the region of chromosome 15, which is involved in Angelman syndrome, were normal. Laboratory investigations excluded an amino acid, organic acid, or a fatty acid *β*-oxidation disorder. However, hypoglycorrhachia (1.5 mmol/L; n.v. 2.8–4.4 mmol/L) with abnormal cerebrospinal fluid (CSF)/blood glucose ratio (0.25; n.v. 0.6) were identified. The CSF lactate concentration was also decreased (0.92 mmol/L; n.v. 1.2–2.1). Despite these results, which are compatible with a glucose transporter type 1 deficiency syndrome (Glut1 DS), no definitive diagnosis was made at the time.

The patient was referred many years later (age 27 years) for a recently developed quasi-fixed cervical dystonia (Figure 1F) without evidence of cervical or brain lesions. Common infectious, metabolic, drug-induced, and autoimmune causes were excluded. Symptomatic treatment with oral anticholinergic biperiden and focal injections of the botulinum toxin allowed for partial relief. The review of her childhood medical records led to the suspicion of Glut1 DS. NGS analysis confirmed this diagnosis by identifying the *de novo* pathogenic variant c.1259T>G in heterozygosity in the *SLC2A1* gene, resulting in the substitution of a highly conserved methionine to an arginine residue in position 420 in the 11th transmembrane segment. A ketogenic diet was initiated. At the 6 month follow-up, the mother of the patient reported a clear improvement in cervical posturing and a disappearance of hyperkinetic appendicular movements.

## 4. Discussion

We present three patients with IEM with different neurological manifestations in whom an accurate diagnosis was achieved through NGS, allowing for personalized treatments.

Patient 1 and her deceased sister would have benefited from an earlier diagnosis and treatment. The beneficial effect of chenodeoxycholic acid on neurological symptoms is considered greater when prescribed earlier in the course of the disease by preventing irreversible damage to the nervous system [10,11]. Interestingly, long diagnostic delays appear to be common in CTX [12]. This may be due to the lack of awareness of this rare disorder and to the variability in its phenotype. For example, in the spinal form of CTX, xanthomas and other typical features may be absent [13]. Moreover, depending on the health system, refusal of medical insurance providers to cover the costs of genetic testing because of the alleged lack of therapeutic consequences may delay the diagnostic process. Taken together, shortening the time to diagnosis is challenging in a field constantly growing in complexity. For this reason, we think that the diagnostic process requires a close collaboration between a multidisciplinary team of physicians who are familiar with rare diseases, including clinical geneticists, metabolic specialists, neurologists, neuroradiologists, and ophthalmologists. In addition, genetic databases and websites, as well platforms for data sharing like Matchmaker Exchange, have become resources at least as important as direct interactions with renowned experts in specific fields [14,15]. Finally, the awareness of clinical, biological, and radiological red flags and symptom constellations suggestive of a treatable neurogenetic disorder will contribute to reducing the time to diagnosis [2]. For example, chronic diarrhea, juvenile cataracts, or tendon xanthomas in patients with progressive neurological symptoms strongly point to CTX, as highlighted by Mignarri et al. [12].

In patient 3, the diagnosis of Glut1 DS was delayed for decades, thereby preventing the implementation of a ketogenic diet, a treatment shown to be effective in controlling seizures and improving movement disorders [9,16,17]. This diagnostic wavering may be explained by the sparse medical knowledge about this newly described syndrome in the early 1990s when most of the investigations were conducted [18,19]. This illustrates the necessity of periodical re-evaluations of patients with unexplained neurologic disorders. Medical knowledge in clinical genetics is constantly progressing, driven by rapidly evolving diagnostic technologies. However, long diagnostic processes may be challenging in adults with an early-onset neurodevelopmental disorder, as this category of patients is often unable to seek medical attention by themselves and may receive limited attention from the community. Consequently, they tend to be less thoroughly investigated than children, which also implies that medical knowledge about the natural history and late-onset complications of rare childhood-onset syndromes is often missing. Atypical adult presentation different from the textbook phenotype defined in children may lead to a missed diagnosis [20].

In patient 2, the clinical phenotype suggested hereditary spastic paraplegia, a large group of diseases encompassing dozens of overlapping entities [21]. NGS was instrumental to the diagnosis of X-ALD in this patient who had normal CNS imaging and no clinical clues to this particular diagnosis prior to genetic testing. This diagnosis allowed for the initiation of corticosteroid replacement therapy, which may prevent a potential adrenal insufficiency crisis. Moreover, allogenic hematopoietic stem cell transplantation might be indicated in patients developing the cerebral form of the disease [22].

In all these cases, the availability and rapidity of NGS allowed for the confirmation of the diagnosis. The genotype-first diagnostic process has become common with the recent advances and increasing availability in clinical settings of genetic technologies, including NGS and DNA microarray, bringing with it greater opportunities for diagnosing patients and multiple family members [23,24]. Complementary to the classic phenotype to genotype method, this approach in particular is indispensable in diagnosing atypical forms of a given disease or conditions characterized by unspecific clinical features, such as cognitive deficiency or autism. However, it poses the challenge of data interpretation knowing that the NGS analysis of large gene panels can lead to the identification of variants of unknown significance and incidental findings [8,25]. Current knowledge and international guidelines for variant interpretation are helpful but still insufficient to remove a large amount of noise when attempting to assign significance to particular findings [8,26].

Our case studies illustrate typical medical trajectories encountered by rare-disease patients due to the complexity of their condition, but also the potential for improvement in diagnosis delay and care due to expanding genetic technologies and awareness of this field within the medical community. Although the increasing amount of available genetic data from control populations and patients, together with the sophistication of diagnostic tools, will facilitate the diagnostic process in the future, the development of emerging therapies will further increase the importance of quick and accurate molecular diagnosis.

## Data Availability

The data presented in this study are available on request from the corresponding author.
